# Dataset of water activity measurements of alcohol:water solutions using a Tunable Diode Laser

**DOI:** 10.1016/j.dib.2017.04.017

**Published:** 2017-04-21

**Authors:** Matthew Allan, Lisa J. Mauer

**Affiliations:** Department of Food Science, Purdue University, 745 Agriculture Mall Drive, W. Lafayette, IN 47907, USA

**Keywords:** Water activity, Alcohol solutions

## Abstract

The data presented in this article are related to the research article entitled “RH-temperature phase diagrams of hydrate forming deliquescent crystalline ingredients” (Allan and Mauer, 2017) [1]. The data are water activity measurements of alcohol:water solutions (methanol:water and ethanol:water solutions at varying molar ratios) at different temperatures collected using the Tunable Diode Laser by Decagon Devices. The measured water activities of ethanol:water solutions were correlated to the initial volumetric ratios to produce polynomial equations that can be used to calculate the needed initial volumetric ratios for water activity controlled solutions. The data sets and polynomial equations are provided to enable extended analyses and applications of the data and calculations for generating and using controlled water activity solutions containing alcohol. An example application of these data is described in the research article mentioned above.

**Specifications Table**

**Table:** 

Subject area	Food Chemistry
More specific subject area	*Physical food chemistry, water activity, phase diagrams, materials science*
Type of data	*Tables, Figures*
How data was acquired	*Water activity measurements collected using a Tunable Diode Laser instrument (Decagon Devices, Pullman, WA)*
Data format	*Raw, Analyzed*
Experimental factors	*Volumetric ratios of alcohols and water were mixed, equilibrated, and the water activities were measured using the Tunable Diode Laser by Decagon Devices.*
Experimental features	*The effects of alcohol type, molar ratio, and temperature on the measured water activity of alcohol-water solutions*
Data source location	*Purdue University Department of Food Science, West Lafayette, Indiana, USA*
Data accessibility	*The data are available within this article*

**Value of the data**

• The water activities of alcohol solutions were measured using a new method that is able to measure the water activity in the presence of volatiles.
• The validity of the Tunable Diode Laser method is supported because the measured water activities are comparable to values previously reported of similar solutions.
• Polynomial equations were generated from the data to calculate initial volumetric ratios of ethanol–water solutions to produce precise water activity controlled solutions.
• Temperature appears to have a minor effect on the water activity of ethanol–water solutions at 20, 25, and 30 °C, and thus the data should be applicable to a range of temperatures.
• This data allows other researchers to generate water activity controlled solutions to extend their applications.

## 1 Data

**Fig. 1:**
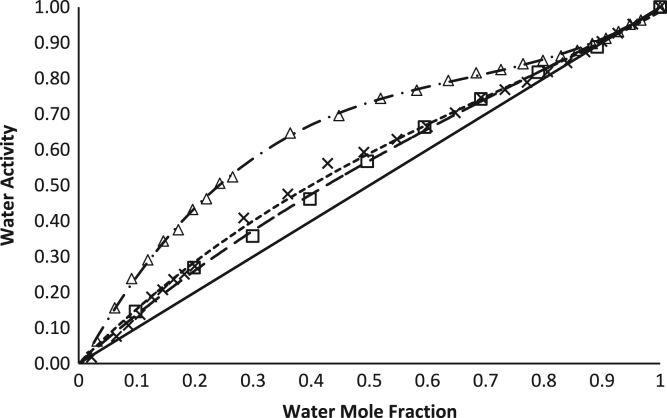
The water activities at 25 °C of water:ethanol solutions measured by the Tunable Diode Laser (─ • Δ ─ •) (numerical data in Table 1), water:methanol solutions measured by the Tunable Diode Laser (─ ─□─ ─) (numerical data in Table 2), the calculated water activities of water:methanol solutions (---×---) from Gӧlles [2] reported by Zhu and others [3], and the water activity of ideal solutions (───) calculated using Raoult׳s law [4].

**Fig. 2:**
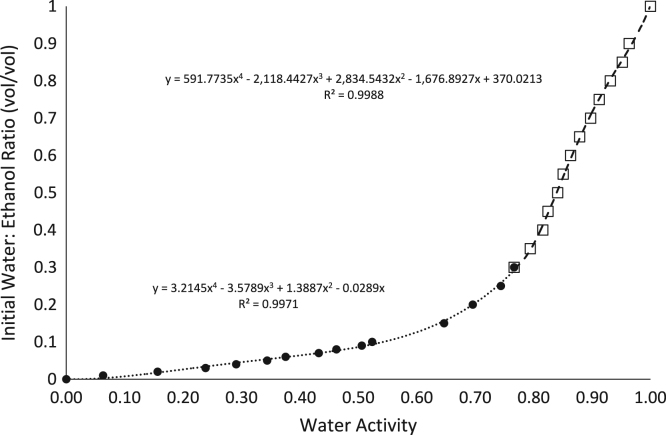
The correlation between the initial volumetric ratio of water to ethanol versus the equilibrated water activity, as measured using the Tunable Diode Laser (numerical data in Table 1). Separate equations were used to determine the relationships between water:ethanol ratios and water activity for solutions below 0.75 water activity (•) and above 0.75 water activity (□) for better accuracy.

**Fig. 3:**
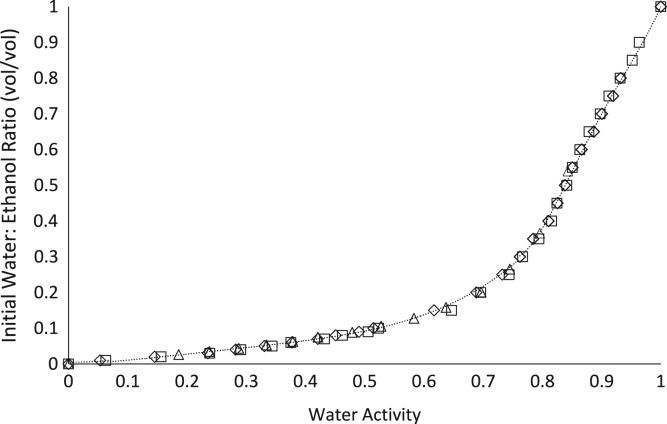
The water activities of water:ethanol solutions at 20 °C (♢), 25 °C (□), and 30 °C (Δ) as measured by the Tunable Diode Laser.

The water activities of alcohol:water solutions were measured using a new water activity (*a*_w_) measurement method, and all data are provided in Table 1, Table 2. The water activities of ethanol:water and methanol:water solutions at varying molar ratios were compared to the reported calculated *a*_w_ methanol:water solutions [2], [3] and the water activity of an ideal solution according to Raoult׳s law [4] in Fig. 1. Molar ratios were converted to initial volumetric ratio then compared to the measured water activity (Fig. 2, and Table 1, Table 2). Polynomial equations were generated to calculate the volumetric ratio of ethanol to water to make water activity-controlled solutions as mentioned in *RH-temperature phase diagrams of hydrate forming deliquescent crystalline ingredients* [1]. The effects of temperature on the water activity of ethanol:water solutions are shown in Fig. 3.

## 2 Experimental design, materials and methods

Ethanol:water and methanol:water solutions were mixed at periodic volumes, equilibrated overnight, and the water activity was measured. Due to the volatility of the alcohols, the water activity of the solutions was measured using the Tunable Diode Laser a_w_ measurement device operating with software version S4TDL-R2-12 (Decagon Devices, Pullman, WA). The Tunable Diode Laser measures the water activity (relative humidity in the headspace above the sample) using a spectroscopic technique at a wavelength unique to water. Before water activity measurements were collected at a given temperature, a multi-point calibration curve was generated using 0.25, 0.50, 0.76, and 1.00 water activity standards from the manufacturer. In addition, the Tunable Diode Laser accuracy was confirmed daily using the same water activity standards plus a 0.92 water activity standard per the manufactures recommendations [5].

The measured water activities were plotted against the volumetric ratios to produce polynomial equations for making water activity-controlled solutions. Two polynomial equations were generated, above and below the inflection point around 0.75 water activity, to improve the accuracy of the calculation. The molar ratios were calculated using the molecular weight and density values of water at 18.02 g/mol and 1.00 g/mL, while the molecular weight and density values of 100% ethanol were 46.07 g/mol and 0.79 g/mL, respectively [6].

## Figures and Tables

**Table 1:** Water activities of ethanol:water solutions at periodic volumetric ratios and the corresponding molar ratios at 25 °C.

Volumetric ratio		Molar ratio		Water activity
Ethanol	Water	Ethanol	Water	
1.00	0.00	1.00	0.00	0.000
0.99	0.01	0.97	0.03	0.063
0.98	0.02	0.94	0.06	0.156
0.97	0.03	0.91	0.09	0.238
0.96	0.04	0.88	0.12	0.291
0.95	0.05	0.85	0.15	0.344
0.94	0.06	0.83	0.17	0.375
0.93	0.07	0.80	0.20	0.432
0.92	0.08	0.78	0.22	0.462
0.91	0.09	0.76	0.24	0.506
0.90	0.10	0.74	0.26	0.524
0.85	0.15	0.64	0.36	0.647
0.80	0.20	0.55	0.45	0.696
0.75	0.25	0.48	0.52	0.744
0.70	0.30	0.42	0.58	0.767
0.65	0.35	0.36	0.64	0.794
0.60	0.40	0.32	0.68	0.816
0.55	0.45	0.27	0.73	0.825
0.50	0.50	0.24	0.76	0.841
0.45	0.55	0.20	0.80	0.851
0.40	0.60	0.17	0.83	0.863
0.35	0.65	0.14	0.86	0.879
0.30	0.70	0.12	0.88	0.898
0.25	0.75	0.09	0.91	0.912
0.20	0.80	0.07	0.93	0.931
0.15	0.85	0.05	0.95	0.952
0.10	0.90	0.03	0.97	0.964
0.00	1.00	0.00	1.00	1.000

**Table 2:** Water activities of methanol:water solutions at periodic volumetric ratios and the corresponding molar ratios at 25 °C.

Volumetric ratio		Molar ratio		Water activity
Methanol	Water	Methanol	Water	
1.00	0.00	1.00	0.00	0.000
0.99	0.01	0.98	0.02	0.007
0.97	0.03	0.94	0.06	0.054
0.96	0.04	0.91	0.09	0.086
0.95	0.05	0.89	0.11	0.130
0.94	0.06	0.87	0.13	0.160
0.93	0.07	0.86	0.14	0.190
0.92	0.08	0.84	0.16	0.215
0.91	0.09	0.82	0.18	0.253
0.90	0.10	0.80	0.20	0.268
0.85	0.15	0.72	0.28	0.395
0.80	0.20	0.64	0.36	0.464
0.75	0.25	0.57	0.43	0.566
0.70	0.30	0.51	0.49	0.602
0.65	0.35	0.45	0.55	0.631
0.60	0.40	0.40	0.60	0.661
0.55	0.45	0.35	0.65	0.694
0.50	0.50	0.31	0.69	0.737
0.45	0.55	0.27	0.73	0.763
0.40	0.60	0.23	0.77	0.785
0.35	0.65	0.19	0.81	0.818
0.30	0.70	0.16	0.84	0.846
0.25	0.75	0.13	0.87	0.881
0.20	0.80	0.10	0.90	0.902
0.15	0.85	0.07	0.93	0.930
0.00	1.00	0.00	1.00	1.000
